# Nuclear Osteopontin Is a Marker of Advanced Heart Failure and Cardiac Allograft Vasculopathy: Evidence From Transplant and Retransplant Hearts

**DOI:** 10.3389/fphys.2020.00928

**Published:** 2020-08-13

**Authors:** Camila Iansen Irion, Julian C. Dunkley, Krista John-Williams, José Manuel Condor Capcha, Serene A. Shehadeh, Andre Pinto, Matthias Loebe, Keith A. Webster, Nicolas A. Brozzi, Lina A. Shehadeh

**Affiliations:** ^1^Interdisciplinary Stem Cell Institute, University of Miami Leonard M. Miller School of Medicine, Miami, FL, United States; ^2^Division of Cardiology, Department of Medicine, University of Miami Leonard M. Miller School of Medicine, Miami, FL, United States; ^3^Department of Pathology, University of Miami Leonard M. Miller School of Medicine, Miami, FL, United States; ^4^Department of Surgery, University of Miami Leonard M. Miller School of Medicine, Miami, FL, United States; ^5^Vascular Biology Institute, University of Miami Leonard M. Miller School of Medicine, Miami, FL, United States; ^6^Peggy and Harold Katz Family Drug Discovery Center, University of Miami Leonard M. Miller School of Medicine, Miami, FL, United States

**Keywords:** heart transplant, cardiac allograft vasculopathy, dilated cardiomyopathy, endomyocardial biopsy, heart retransplantation, osteopontin

## Abstract

**Background:**

Heart transplant is the gold standard therapy for patients with advanced heart failure. Over 5,500 heart transplants are performed every year worldwide. Cardiac allograft vasculopathy (CAV) is a common complication post-heart transplant which reduces survival and often necessitates heart retransplantation. Post-transplant follow-up requires serial coronary angiography and endomyocardial biopsy (EMB) for CAV and allograft rejection screening, respectively; both of which are invasive procedures. This study aims to determine whether osteopontin (OPN) protein, a fibrosis marker often present in chronic heart disease, represents a novel biomarker for CAV.

**Methods:**

Expression of OPN was analyzed in cardiac tissue obtained from patients undergoing heart retransplantation using immunofluorescence imaging (*n* = 20). Tissues from native explanted hearts and three serial follow-up EMB samples of transplanted hearts were also analyzed in five of these patients.

**Results:**

Fifteen out of 20 patients undergoing retransplantation had CAV. 13/15 patients with CAV expressed nuclear OPN. 5/5 patients with multiple tissue samples expressed nuclear OPN in both 1^*st*^ and 2^*nd*^ explanted hearts, while 0/5 expressed nuclear OPN in any of the follow-up EMBs. 4/5 of these patients had an initial diagnosis of dilated cardiomyopathy (DCM).

**Conclusion:**

Nuclear localization of OPN in cardiomyocytes of patients with CAV was evident at the time of cardiac retransplant as well as in patients with DCM at the time of the 1^*st*^ transplant. The results implicate nuclear OPN as a novel biomarker for severe CAV and DCM.

## Introduction

Heart transplant is the gold standard therapy for patients with advanced heart failure. Over 5,500 heart transplants are performed every year worldwide (Khush et al., 2019). Heart transplantation improves patient survival but is accompanied by significant complications and comorbidities that can compromise outcome over time. One such complication, cardiac allograft vasculopathy (CAV), significantly reduces survival when it occurs within 3 years of post-transplant (Khush et al., 2019) and is the most common indication for heart retransplantation (Goerler et al., 2008). CAV is an accelerated fibroproliferative disease (Chih et al., 2016) affecting the vessels of the transplanted heart. The pathology of CAV differs from typical coronary artery disease (CAD). Whereas intimal plaque formation in CAD is focal, eccentric, and typically confined to proximal coronary arteries, in CAV there is diffuse concentric plaque formation affecting all vessels in the allograft, including the veins (Ramzy et al., 2005). Additionally, CAD involves calcium deposition and rarely shows signs of inflammation, while CAV rarely has calcium deposition and is usually associated with inflammation (Aranda and Hill, 2000). CAV, through progressive myocardial ischemic injury, eventually leads to chronic cardiac graft failure (CGF) similar to the contribution of CAD to congestive heart failure (Khan et al., 2009), and this, in turn, necessitates retransplantation (Johnson et al., 2007).

The current mainstay of CAV screening involves serial coronary angiography (Stehlik et al., 2018), an invasive procedure that underestimates the disease burden (Almufleh et al., 2020). Non-invasive biomarkers for CAV including serum C-reactive protein (Stehlik et al., 2018), brain natriuretic peptide (Mehra et al., 2004), troponin I (Colvin-Adams and Agnihotri, 2011), microRNA 628-5p (Neumann et al., 2017), and circulating apoptotic endothelial cells (Singh et al., 2012) have been proposed but none have progressed to clinical application as screening tools. Post-cardiac transplant follow-up also includes screening for allograft rejection, the gold standard for which is serial endomyocardial biopsy (EMB) which is also invasive (Strecker et al., 2013).

Many patients with CAV have microvasculopathy seen on routine EMB. This, however, is not used for diagnosis because it is considered low sensitivity due to the exclusion of microvasculature in many EMBs. Current data suggest that the role of identifying microvasculopathy on EMB in CAV is more relevant to prognosis than to diagnosis (Hiemann et al., 2007). The discovery of a reliable invasive biomarker in cardiac biopsy tissue could allow for concurrent CAV/graft rejection screening without subjecting patients to additional procedures.

Osteopontin (OPN) is a multifunctional secreted glycoprotein synthesized by multiple cells and tissues (Lund et al., 2009). OPN is a pro-inflammatory cytokine that plays a role in the recruitment of immune cells and type-1 (Th1) cytokine expression to areas of inflammation (Singh et al., 2014). It is implicated in the pathogenesis of multiple disease states including atherosclerosis and chronic inflammatory disorders such as Crohn’s disease, lupus, rheumatoid arthritis, and multiple sclerosis. Under normal circumstances, OPN expression in the heart is low (Singh et al., 2014); however, plasma OPN has been used as a causative biomarker for various cardiac pathologic states such as myocardial infarction (Trueblood et al., 2001), left ventricular hypertrophy (Graf et al., 1997), and heart failure (Stawowy et al., 2002; Li et al., 2017; Yousefi et al., 2019). OPN was recently proposed as a biological marker and prognostic indicator of chronic ischemic heart disease (Coculescu et al., 2019). Considering the significant role of OPN not only in inflammatory and fibrotic processes as well as several cardiac pathologies, it comes as no surprise that OPN may be implicated in CAV.

In order to determine if OPN could serve as a novel biomarker for CAV, in this retrospective study we analyzed the expression pattern of OPN in cardiac tissue from 20 heart transplant patients receiving retransplantation. For 5 of these 20 patients, we also analyzed tissues from the native explanted hearts as well as three serial follow-up biopsy samples of the transplanted hearts.

## Materials and Methods

### Myocardial Tissue Samples

Cardiac tissue samples from 20 patients subjected to heart retransplantation were obtained from the Department of Pathology at the, Miller School of Medicine, University of Miami. The ages of the patients ranged between 7 and 69 years at the time of the 2^*nd*^ heart transplant. Each patient underwent heart retransplantation surgery at our institution between 2005 and 2019. Table 1 delineates the demographic data and clinical conditions of all individual patients used for this study.

**TABLE 1:** Demographic data and clinical condition of individual patients at time of 2^*nd*^ heart transplant, as reflected by NYHA CF classification (I–IV).

**Patient #**	**Diagnosis**	**Time since 1^*st*^ heart Tx (years)**	**Clinical condition**	**Comments**
1	CGF	7	IV	Chronic transplant coronary artery vasculopathy
2	AMR	9	IV	Recurrent AMR and kidney failure
3	PGF	2 days	IV (shock / ECMO)	1^*st*^ heart allograft removed within 48 h and insertion TAH Thoratec
4	CGF	10	IV	Chronic transplant coronary artery vasculopathy; developed renal failure
5	CGF	11	II-III	Chronic transplant coronary artery vasculopathy
6	CGF	17	IV	Chronic transplant coronary artery vasculopathy
7	PGF	9 days	IV (shock / ECMO)	1st heart allograft removed within 9 days
8	CGF	16	IV	Chronic transplant coronary artery vasculopathy
9	CGF	8	IV	Chronic transplant coronary artery vasculopathy
10	RHF	14	III	Constrictive pericarditis (symptoms of right heart failure)
11	CGF	10	IV (shock / ECMO)	Chronic transplant coronary artery vasculopathy
12	CGF	14	IV	Chronic transplant coronary artery vasculopathy
13	CGF	11	III-IV	Chronic transplant coronary artery vasculopathy
14	CGF	13	IV	Chronic transplant coronary artery vasculopathy
15	CGF	6	II-III	Chronic transplant coronary artery vasculopathy
16	CGF	13	IV (shock / BiVentricular support)	Chronic transplant coronary artery vasculopathy, progressed to cardiogenic shock. Heart explant and insertion TAH Thoratec
17	CGF	13	III	Chronic transplant coronary artery vasculopathy
18	CGF	9	III	Chronic transplant coronary artery vasculopathy
19	CGF	9	II-III	Chronic transplant coronary artery vasculopathy
20	CGF	6	IV	Diastolic heart failure(symptoms of right heart failure)

*CGF, chronic graft failure; AMR, antibody mediated rejection; PGF, primary graft failure (acute graft failure); RHF, right heart failure; Tx, transplantation; TAH, total artificial heart; ECMO, extracorporeal membrane oxygenation. Clinical condition of the patient is reflected in 4 tier scale, as by NYHA functional classification: I = asymptomatic, II = minimal symptoms on exertion, III = symptoms on moderate efforts, IV = symptoms at rest or with minimal exercise.*

For patients 1–5, we received samples from the 1st explant, follow-up biopsies between the heart transplants, and lastly the 2nd explant. The first biopsy sample for each patient was taken 8–16 days post-transplant. The second biopsy was taken around a month after the first biopsy. The third biopsy was taken 1 year after the transplant. For one of these five patients, the first heart allograft was removed within 48 h due to primary graft failure (PGF). For this reason, only one biopsy was done 1 day after the surgery before retransplantation. Table 2 presents relevant clinical information for these five patients.

**TABLE 2:** Clinical condition of individual patients at time of myocardial biopsy, as reflected by NYHA CF classification (I–IV).

**Patient #**	**Explant # #1**	**Biopsy #1**	**Biopsy # 2**	**Biopsy # 3**	**Explant # 2**	**Comments**
1 (Dx = VCM)	IV	II (10 days)	I (1 month)	I (1 year)	IV (7 years)	Chronic rejection led to 2^*nd*^ heart Tx
2 (Dx = VCM)	IV	II (8 days)	I (1 month)	I (3 years)	IV (9 years)	Recurrent AMR and kidney failure
3 (Dx = DCM)	IV	Shock (1 day)	Shock (2 days)	-	-	1^*st*^ Heart Tx removed within 2 days
4 (Dx = DCM)	IV	II (12 days)	I (5 weeks)	II (1 year)	IV (11 years)	Developed renal failure
5 (Dx = DCM)	IV	I (16 days)	I (6 weeks)	I (1 year)	II–III (9 years)	Chronic coronary artery vasculopathy

*Dx, diagnosis; VCM, valvular cardiomyopathy; DCM, dilated cardiomyopathy; AMR, anti-body mediated rejection requiring plasmapheresis and thymoglobulin; Tx, transplantation; Explant # 1, pathology of native heart explanted at time of 1^*st*^ heart transplant; Biopsy, endomyocardial biopsy after 1^*st*^ heart transplant; Explant # 2, pathology of 1st transplanted heart, obtained at time of 2^*nd*^ heart transplant.*

We also analyzed myocardial biopsy samples from three additional patients who underwent heart transplant and developed post-transplant infectious complications leading to sepsis. Demographic information pertaining to these patients is shown in Supplementary Table S1.

This retrospective study was carried out in accordance with the recommendations of the University of Miami Institutional Review Board (IRB protocol # 2018-0439). All specimens were de-identified (assigned patients #1–20 and sepsis patients #1–3) and archived prior to the study, therefore negating the need for consent forms.

### Immunofluorescence Staining, Image Acquisition, and Statistical Analyses

**FIGURE 1:**
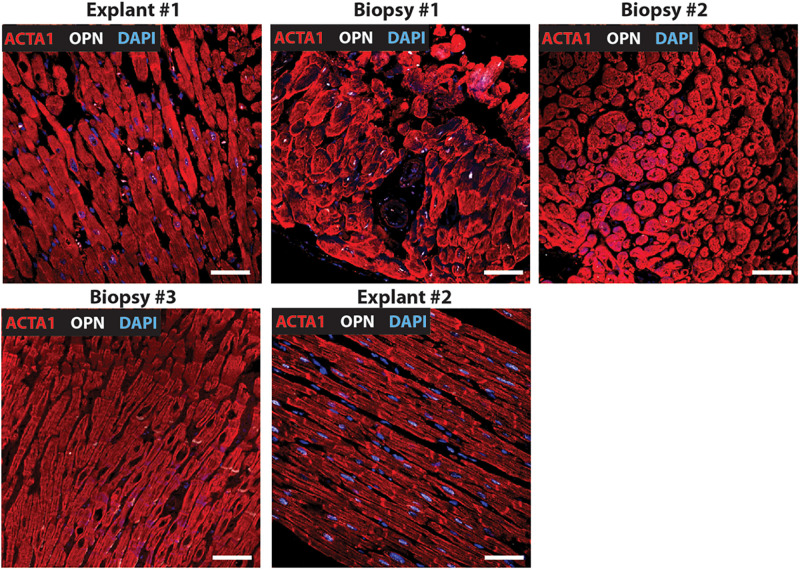
Nuclear OPN expression in explanted hearts but not endomyocardial biopsies (EMBs). In Patient #1, nuclear OPN (shown in white) in ACTA1-positive cardiomyocytes (shown in red) is visible in failing native and transplanted hearts, but in none of the three serial follow-up EMB samples collected at 1 week, 1 month, and 1 year post first transplant. Representative confocal *z*-stack images are shown. Scale bar = 20 μm.

**FIGURE 2:**
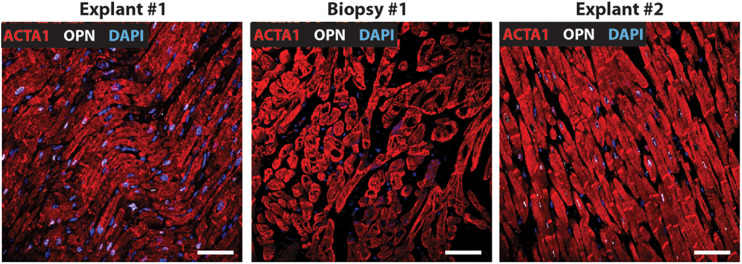
Nuclear OPN expression in explanted hearts but not endomyocardial biopsies (EMBs). In Patient #2, nuclear OPN (shown in white) in ACTA1-positive cardiomyocytes (shown in red) is visible in failing native and transplanted hearts, but not in the serial follow-up EMB sample collected at almost 1 week post first transplant. Representative confocal *z*-stack images are shown. Scale bar = 20 μm.

**FIGURE 3:**
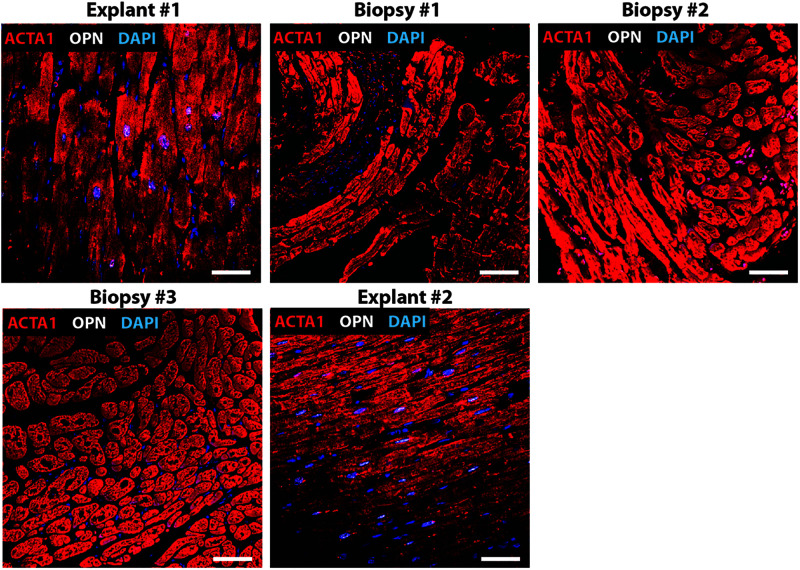
Nuclear OPN expression in explanted hearts but not endomyocardial biopsies (EMBs). In Patient #3, nuclear OPN (shown in white) in ACTA1-positive cardiomyocytes (shown in red) is visible in failing native and transplanted hearts, but in none of the three serial follow-up EMB samples collected at 1 week, 1 month, and 1 year post first transplant. Representative confocal *z*-stack images are shown. Scale bar = 20 μm.

**FIGURE 4:**
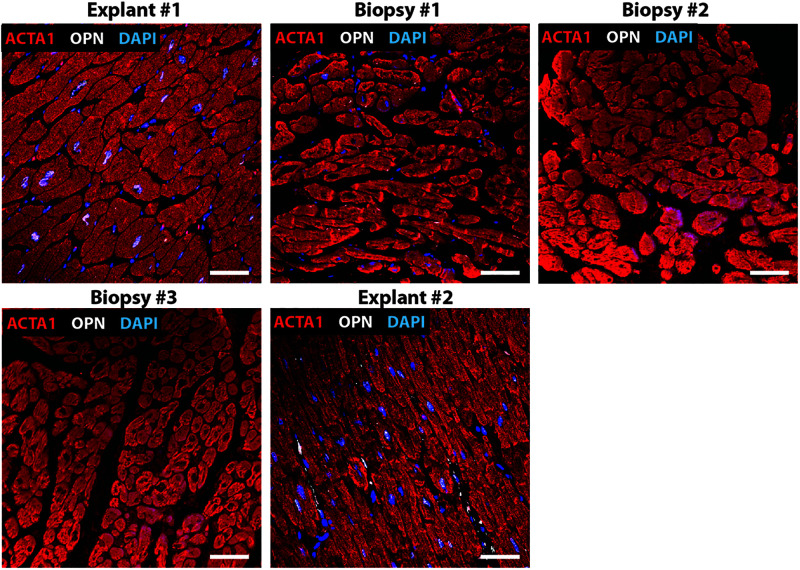
Nuclear OPN expression in explanted hearts but not endomyocardial biopsies (EMBs). In Patient #4, nuclear OPN (shown in white) in ACTA1-positive cardiomyocytes (shown in red) is visible in failing native and transplanted hearts, but in none of the three serial follow-up EMB samples collected at 1 week, 1 month, and 1 year post first transplant. Representative confocal *z*-stack images. Scale bar = 20 μm.

**FIGURE 5:**
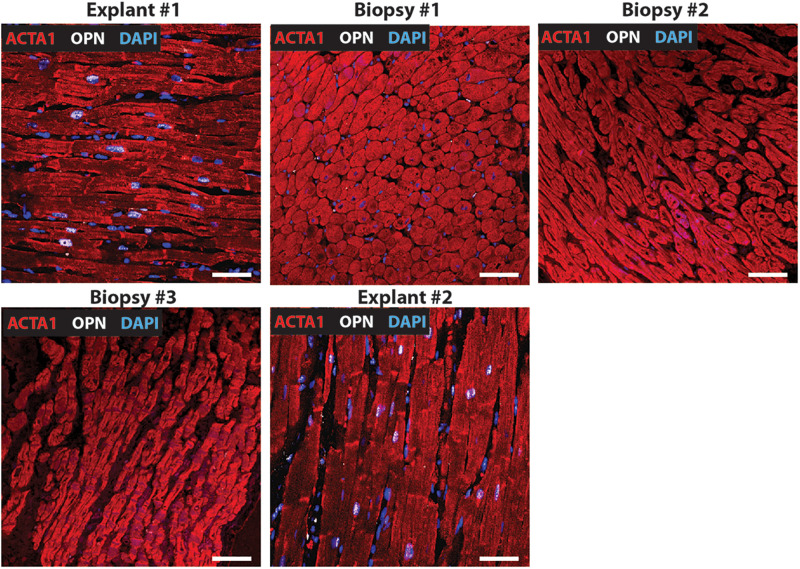
Nuclear OPN expression in explanted hearts but not endomyocardial biopsies (EMBs). In Patient #5, nuclear OPN (shown in white) in ACTA1-positive cardiomyocytes (shown in red) is visible in failing native and transplanted hearts, but in none of the three serial follow-up EMB samples collected at 1 week, 1 month, and 1 year post first transplant. Representative confocal *z*-stack images are shown. Scale bar = 20 μm.

To investigate the presence of OPN in paraffin-embedded sections, immunofluorescence imaging was performed in heart tissues from all patients as previously described (Irion et al., 2018). Three slides per heart were stained and five to six images per slide were analyzed. In brief, samples were incubated for 45 min at 70°C, dewaxed with two 5-min xylene washes, and hydrated by 3-min graded ethanol washes of 100% (twice), 95%, 80%, and 70% followed by two 4-min water immersions. Antigen retrieval was achieved by steaming the slides for 75 min in 1× Citrate Antigen Retrieval Buffer (Ab93678). Samples were permeabilized with 0.2% Triton X-100 (Sigma-Aldrich) for 30 min, followed by blocking with 10% Donkey serum in TBST for 45 min. Slides were incubated with primary antibodies against Alpha Skeletal Muscle Actin, Alpha-Sr 1 (ACTA-1) (Abcam Ab28052; dilution 1:200), and hOPN (R&D AF1433; dilution 1:50) overnight at 4°C. ACTA-1 was used as a cardiomyocyte marker. Slides were washed with PBS and then incubated for 1 h at room temperature with conjugated secondary antibodies: Donkey anti-Mouse IgG, Alexa Fluor 568 (Thermo Fisher Scientific A-10037; 1:400) and Donkey anti-Goat IgG, Alexa Fluor 647 (Thermo Fisher Scientific A-21447, 1:400) in blocking solution. DAPI was used to stain nuclei before mounting with ProLong Gold Antifade (Invitrogen P36934) and coverslips. To determine myocyte area, paraffin-embedded sections were stained with Alexa Fluor 555 Wheat Germ Agglutinin (WGA) (Thermo Fisher Scientific W32464) as recommended by the manufacturer’s instructions to stain cell membrane. All slides were scanned at 20× magnification using an Olympus VS120–L100 Virtual Slide Microscope (Tokyo, Japan) and analyzed using Olympus OlyVIA 2.9 software. Representative confocal images (shown in Figures 1–5) were captured at 40× magnification and 0.6× digital zoom using Z-stacking on a Zeiss LSM710 confocal microscope.

A quantitative analysis was performed by two investigators to determine the number of cells including cardiomyocytes and non-cardiomyocyte with nuclear OPN in representative fields. Six images per sample were counted and averaged from all 2^*nd*^ explants. Values were expressed in percentage (%). Myocyte area (μm^2^) was measured in Image J using WGA images from all 2^*nd*^ explants. Five WGA images per sample were used and at least 30 cells per image were analyzed by three investigators. For statistical analysis, unpaired *t*-test was used for comparing two groups, and one-way ANOVA with Tukey’s *post hoc* test was used for comparing more than two groups. A linear correlation between myocyte area and % of myocyte cells with nuclear OPN was applied. Data are presented as mean ± SD and *p*-values < 0.05 were considered significant. Statistical analyses were performed with Prism 7.05 software (GraphPad Software Inc, San Diego, CA, United States).

## Results

Immunofluorescence was performed on paraffin-embedded sections of myocardial explants, and biopsies obtained from heart transplant patients to evaluate the presence of OPN. Out of a total of 20 patients who received 2^*nd*^ transplant, 15 had CAV. Of these 15 CAV patients, 13 patients expressed nuclear OPN (86.67%). In the 5 non-CAV patients, 4 (80%) expressed nuclear OPN. The overall clinical diagnoses included primary graft failure, antibody-mediated rejection, constrictive pericarditis with right heart failure, and diastolic heart failure with symptoms of right heart failure. Of these, the single patient presenting without CAV or nuclear OPN expression had primary graft failure with allograft removal only after 9 days. The single patient with antibody-mediated rejection expressed OPN in the nucleus despite receiving the 2^*nd*^ transplant only 2 days after the first transplanted heart. Individual immunofluorescence images for each of the 20 patients are displayed in Supplementary Figure S1. As shown in Figures 1–5, all five patients presented with nuclear OPN in both their 1st and 2nd explants. In the serial biopsies, however, no OPN expression was seen; 4/5 of these patients had diagnoses of dilated cardiomyopathy and 3/5 had CAV at the time of retransplantation.

Interestingly, patient #7 developed primary graft failure (heart was removed 9 days after transplant) and did not express OPN in the nucleus indicating that OPN expression may only be present after long-standing disease. When we analyzed the heart tissue from sepsis patients, all three patients expressed OPN in the nucleus and one patient also expressed OPN in the cytoplasm as seen in Supplementary Figure S2.

**FIGURE 6:**
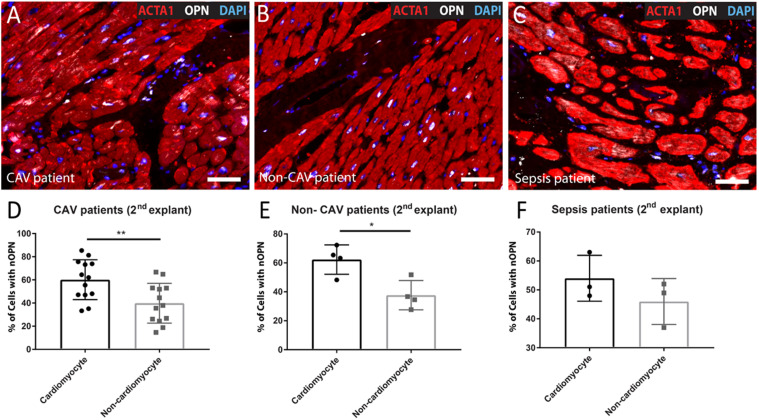
Representative images of 2^*nd*^ explant hearts from CAV **(A)**, non-CAV **(B)**, and sepsis patients **(C)** showing the presence of OPN in the nucleus in cardiomyocytes and non-cardiomyocytes. **(D)** Percentage of cardiomyocytes and non-cardiomyocytes with nuclear OPN expression in 2^*nd*^ explant hearts from patients with CAV (*n* = 13) and **(E)** non-CAV patients (*n* = 4). In both cases, cardiomyocytes presented a higher percentage of nuclear OPN compared to non-cardiomyocytes. No differences were observed in patients with sepsis **(F)**. Unpaired *t*-test. * *p* < 0.05, **p < 0.01. CAV, cardiac allograft vasculopathy; nOPN, nuclear OPN. Scale bar = 20 μm.

**FIGURE 7:**
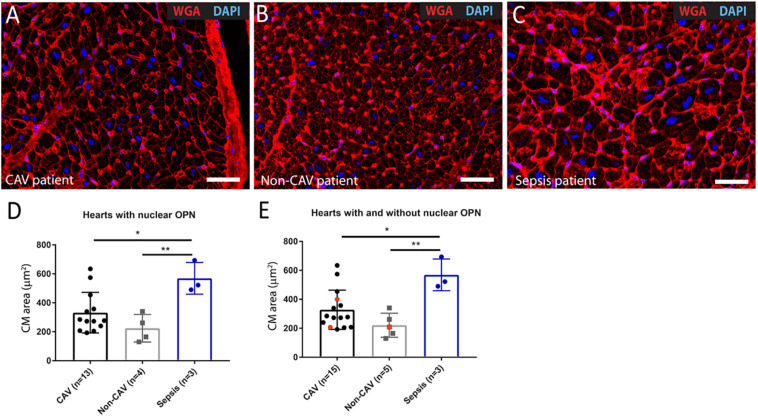
Representative images of 2^*nd*^ explant hearts from CAV **(A)**, non-CAV **(B)**, and sepsis patients **(C)** showing staining with WGA-555 to evaluate myocyte area. Patients with sepsis presented a significant increase in myocyte area when compared to CAV and non-CAV patients in hearts with nuclear OPN **(D)** and all hearts with and without nuclear OPN **(E)**. The few hearts without nuclear OPN are indicated in red. One-way ANOVA with Tukey’s *post hoc* test, *p < 0.05, **p < 0.01. CAV, cardiac allograft vasculopathy; CM, cardiomyocyte; WGA, Wheat Germ Agglutinin. Scale bar = 20 μm.

**FIGURE 8:**
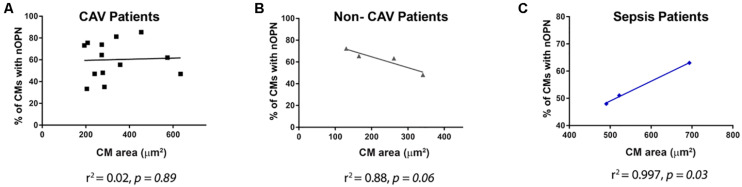
Correlations between myocyte area and % of cardiomyocytes with nuclear OPN in CAV **(A)**, non-CAV **(B)**, and sepsis **(C)** patients are shown. Pearson’s correlation *p < 0.05. CAV, cardiac allograft vasculopathy; CM, cardiomyocyte; nOPN, nuclear OPN.

When we evaluated the percentage of cells, including cardiomyocytes and non-cardiomyocytes that express OPN in the nucleus in the 2^*nd*^ explant, both CAV patients and non-CAV patients had a higher percentage of cardiomyocytes expressing OPN in the nucleus than non-cardiomyocytes cells (CAV patients: 60.14% ± 17.22 of cardiomyocytes with nuclear OPN vs. 39.86% ± 17.22 of non-cardiomyocytes with nuclear OPN, *p* = 0.0062; non-CAV patients: 62.31% ± 10.16 of cardiomyocytes with nuclear OPN vs. 37.69% ± 10.15 of non-cardiomyocytes with nuclear OPN, *p* = 0.014) as seen in Figures 6A,B,D,E. In patients with sepsis, this difference was not significant however, the results followed a similar trend (Figures 6C,F). Patients with sepsis presented a significant increase in myocyte area when compared with CAV and non-CAV groups (*p* < 0.05) as shown in Figure 7. On the other hand, in CAV patients, there was a trend for increased myocyte area compared with non-CAV patients (Figure 7). These findings were noted in all patients regardless of the presence or absence of OPN expression in the cardiomyocyte nuclei. No significant correlation between myocyte area and % of cells with OPN in the nucleus was present in CAV and non-CAV patients; however, in sepsis patients, a positive correlation was observed (Figure 8).

## Discussion

Heart retransplantation accounts for 2–4% of heart transplants performed every year in the United States and has remained constant over the years. Major indications for cardiac retransplantation include early graft failure and coronary vasculopathy (Lund et al., 2014). Although the overall survival of retransplant patients is lower than that of first transplants (70% at 1 year, 55% at 5 years vs. 85% at 1-year, 69% at 5-years), subjects who are retransplanted more than 1 year after the first transplant have significantly higher survival rates than those transplanted within 1 year of the first transplant (unadjusted Hazard Ratio 1.79, *p* < 0.001) (Miller et al., 2019). Therefore, it is important to develop diagnostic procedures to identify those cardiac transplant patients with CAV and/or early graft failure who are high-risk candidates for early retransplantation.

Here, we show for the first time, the presence of nuclear OPN in human myocardial tissue from explanted hearts taken after heart retransplantation. The presence of nuclear OPN in immunofluorescence imaging of cardiac biopsies has the potential to act as an invasive biomarker for CAV and retransplantation. Our results showed that 13/15 patients with CAV had clear OPN expression in cardiomyocyte nuclei. If OPN is proven to have a role in this pathogenesis, it may also serve as a novel therapeutic target for which blockade may delay or prevent the further development of this condition. The possibility also exists that nuclear OPN localization may occur concurrently with or prior to CAV development in transplant patients. This could be applied to CAV screening during endomyocardial biopsy in conjunction with coronary angiography or be implemented in the risk stratification of these patients. Whereas other biological markers for CAV have been implicated in various studies (Mehra et al., 2004; Colvin-Adams and Agnihotri, 2011; Singh et al., 2012; Neumann et al., 2017; Stehlik et al., 2018), these have not been applied generally for CAV prediction or as diagnostic screening tools in clinical settings to our knowledge. Whereas we do not know the reasons for this, our present evidence using clinical samples acquired directly from transplant patient samples supports such clinical application for OPN that may be used as alternative or in addition to one or more of these other markers to confirm the condition.

Four out of five patients for whom we analyzed explants x2 and biopsies x3 had diagnoses of idiopathic dilated cardiomyopathy at the time of their first heart transplant. Each of these patients expressed OPN in the nuclei of the cardiomyocytes. These results suggest a strong correlation between end-stage dilated cardiomyopathy and nuclear localization of OPN. Dilated cardiomyopathy (DCM) shows myocyte hypertrophy and interstitial fibrosis on EMB, which are nonspecific but abnormal histopathological features (Cunningham et al., 2006). OPN localization in the nucleus could potentially correlate with severity of DCM and therefore serve as a prognostic biomarker. The usefulness of routine EMB in idiopathic DCM is still under debate (Cooper, 2013), but immunofluorescence imaging for OPN could provide added utility for this test.

Based on staining of the available follow-up cardiac biopsies, we were able to validate the association of cardiomyocyte nuclear OPN localization with a disease state. Whereas at the time of both 1^*st*^ and 2^*nd*^ heart transplantation, explanted hearts showed OPN expression in the nucleus; in 5/5 patients with recently transplanted healthy allografts, OPN was not detected by at any of the biopsy time points (1 week, 1 month, 1 year). Because recently transplanted cardiac allografts are free of chronic heart disease, this consistent with our hypothesis that nuclear OPN is associated with end-stage heart disease requiring heart transplant and retransplant.

Other studies have reported the presence of different OPN isoforms in tumor cells *in vivo* where its presence correlates with tumor aggression, poor prognosis, and enhanced metastasis, at least in part by regulating PI3K/Akt/GSK3β and endothelial-mesenchymal transition (EMT) pathways, perhaps in some cancers through interaction with hypoxia-inducible factors (HIF-1α and 2α) (Gomaa et al., 2013; Jia et al., 2016). Isoforms OPN-a and OPN-b were seen only in the nucleus while OPN-c was seen in the cytoplasm of breast carcinoma cells. OPN-c, present in 75% of breast cancer patients, was absent in normal tissues, while OPN-a and OPN-b were identified in both normal tissues and breast carcinoma cells (Castello et al., 2017). To determine the role of OPN in cell proliferation, fluorescent OPN-GFP fusion proteins were transfected into embryonic kidney 293 cells and revealed nuclear localization of OPN. In the same study, endogenous OPN also stained strongly in nuclei (Junaid et al., 2007). To date, nuclear localization of OPN in cardiomyocytes *in situ* has not been reported.

Our group has previously described the presence of increased OPN expression in cardiomyocyte cytoplasm in sepsis in pediatric patients (Irion et al., 2018), which occurs as a result of widespread inflammation in the body. Of note, there were three additional explant tissue samples taken from patients suffering from sepsis at the time of the transplant surgery (shown in Supplementary Figure S2). OPN was localized to the nucleus in all 3 patients as well as in the cytoplasm for 1/3 patients.

In CAV and non-CAV transplant patients cardiomyocytes presented a higher percentage of nuclear OPN compared to non-cardiomyocytes. Interestingly, myocyte area was higher in sepsis transplant patients compared to CAV and non-CAV transplant patients. Myocyte area was also correlated to percentage of cardiomyocytes with nuclear OPN in sepsis transplant patients, but not in CAV and non-CAV transplant patients.

### Limitations

Serial biopsy samples were available for only 5/20 patients; therefore, for the remaining 15 patients, we could not confirm that OPN nuclear localization was indeed absent during the “chronic disease-free state.” Additionally, EMB samples were limited to 1-year post-transplant so we have no data on the time course of progression of OPN nuclear localization. Whereas 20 patients may be considered a small sample size for a clinical study, cardiac retransplantation is rare and 20 cases from a single center is a significant achievement. However, this is acknowledged as a limitation and additional testing to confirm these preliminary findings are warranted.

## Conclusion

In this study, we report clear nuclear localization of OPN in cardiomyocytes of patients suffering from cardiac allograft vasculopathy at the time of cardiac retransplant as well as patients with dilated cardiomyopathy at the time of the 1^*st*^ transplant. These conditions involve fibrotic processes of the coronary vasculature and myocardium, respectively. This is the first study to report nuclear OPN expression in cardiac tissue *in situ*. Our findings highlight a potentially important role for OPN as a novel biomarker in severe CAV and DCM.

## Data Availability Statement

All datasets presented in this study are included in the article/Supplementary Material.

## Ethics Statement

This retrospective study was carried out in accordance with the recommendations of the University of Miami Institutional Review Board (IRB protocol # 2018-0439). All specimens were de-identified (assigned patients # 1–20) and archived prior to the study, therefore negating the need for consent forms.

## Author Contributions

CI and KJ-W performed the experiments. CI, JD, KJ-W, SS, JC, AP, NB, and LS analyzed the data. CI, JD, KJ-W, SS, JC, AP, KW, NB, and LS interpreted the results of experiments. CI, KW, JC, and LS prepared the figures. CI and JD drafted the manuscript. CI, JD, KW, NB, and LS edited and revised the manuscript. CI, JD, KJ-W, SS, JC, AP, ML, KW, NB, and LS approved the final version of the manuscript. All authors contributed to the article and approved the submitted version.

## Conflict of Interest

The authors declare that the research was conducted in the absence of any commercial or financial relationships that could be construed as a potential conflict of interest.

## Supplementary Material

FIGURE S1 Nuclear OPN expression in failing transplanted hearts. In 15/20 patients undergoing second heart transplant, nuclear OPN (shown in white) in ACTA1 positive cardiomyocytes (shown in red) is visible in the failing transplanted hearts. Shown are representative fluorescence images from scanner microscope. Scale bar = 20 μm.

FIGURE S2 Nuclear OPN expression in failing native hearts of sepsis patients. In 3/3 sepsis patients undergoing a heart transplant, nuclear OPN (shown in white) in ACTA1 positive cardiomyocytes (shown in red) is visible in the failing native hearts. Shown are representative fluorescence images from scanner microscope. Scale bar = 20 μm.

TABLE S1 Clinical condition of three patients with sepsis at time of 1st heart transplant. CAD: coronary artery disease.
