# Supplementary figures and images for: Nuclear Osteopontin Is a Marker of Advanced Heart Failure and Cardiac Allograft Vasculopathy: Evidence From Transplant and Retransplant Hearts
Source: Front Physiol. 2020 Aug 13;11:928. doi: 10.3389/fphys.2020.00928 (PMC7438570; doi:10.3389/fphys.2020.00928)

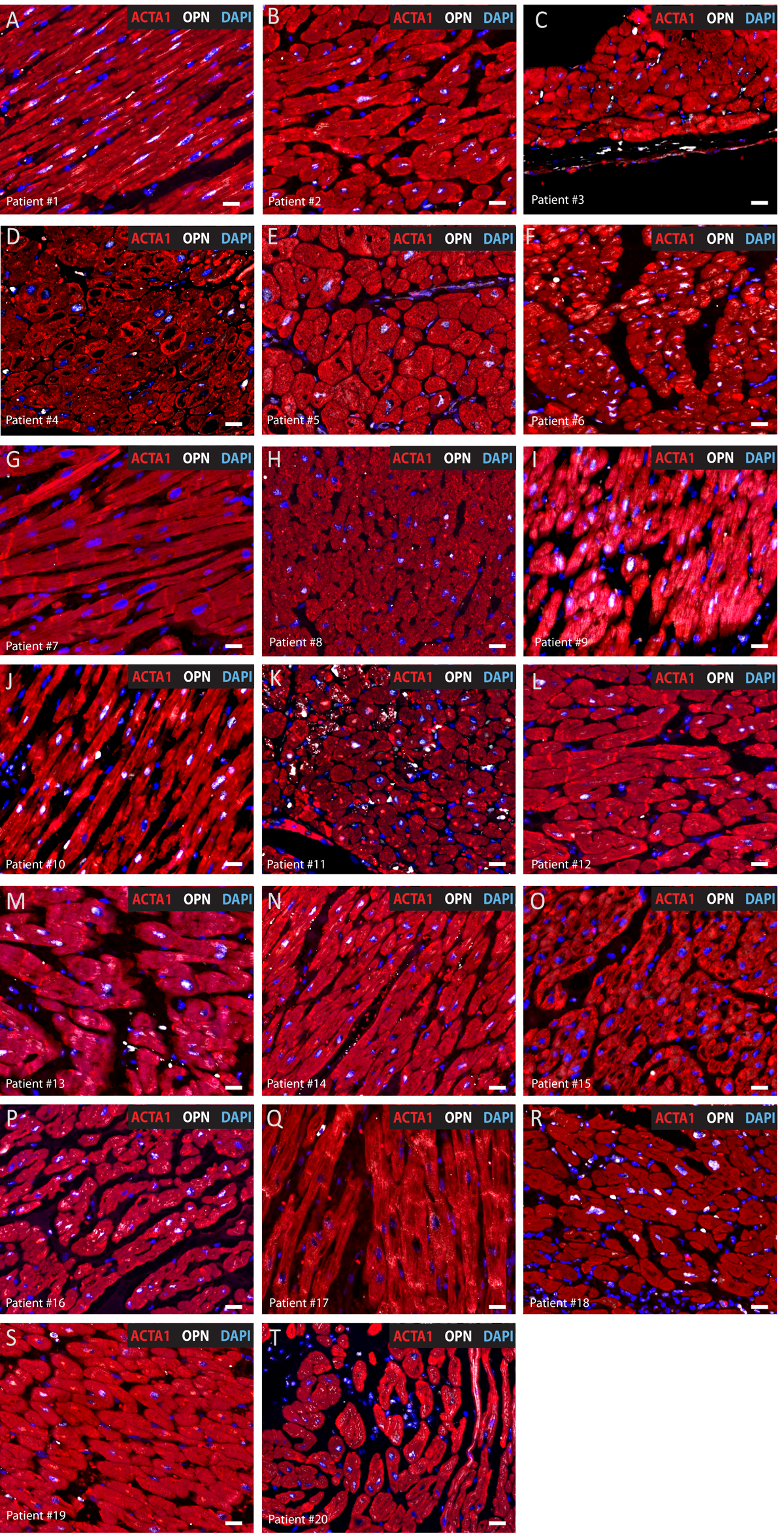

Supplement: FIGURE S1 — Nuclear OPN expression in failing transplanted hearts. In 15/20 patients undergoing second heart transplant, nuclear OPN (shown in white) in ACTA1 positive cardiomyocytes (shown in red) is visible in the failing transplanted hearts. Shown are representative fluorescence images from scanner microscope. Scale bar = 20 μm. [file Image_1.TIF]

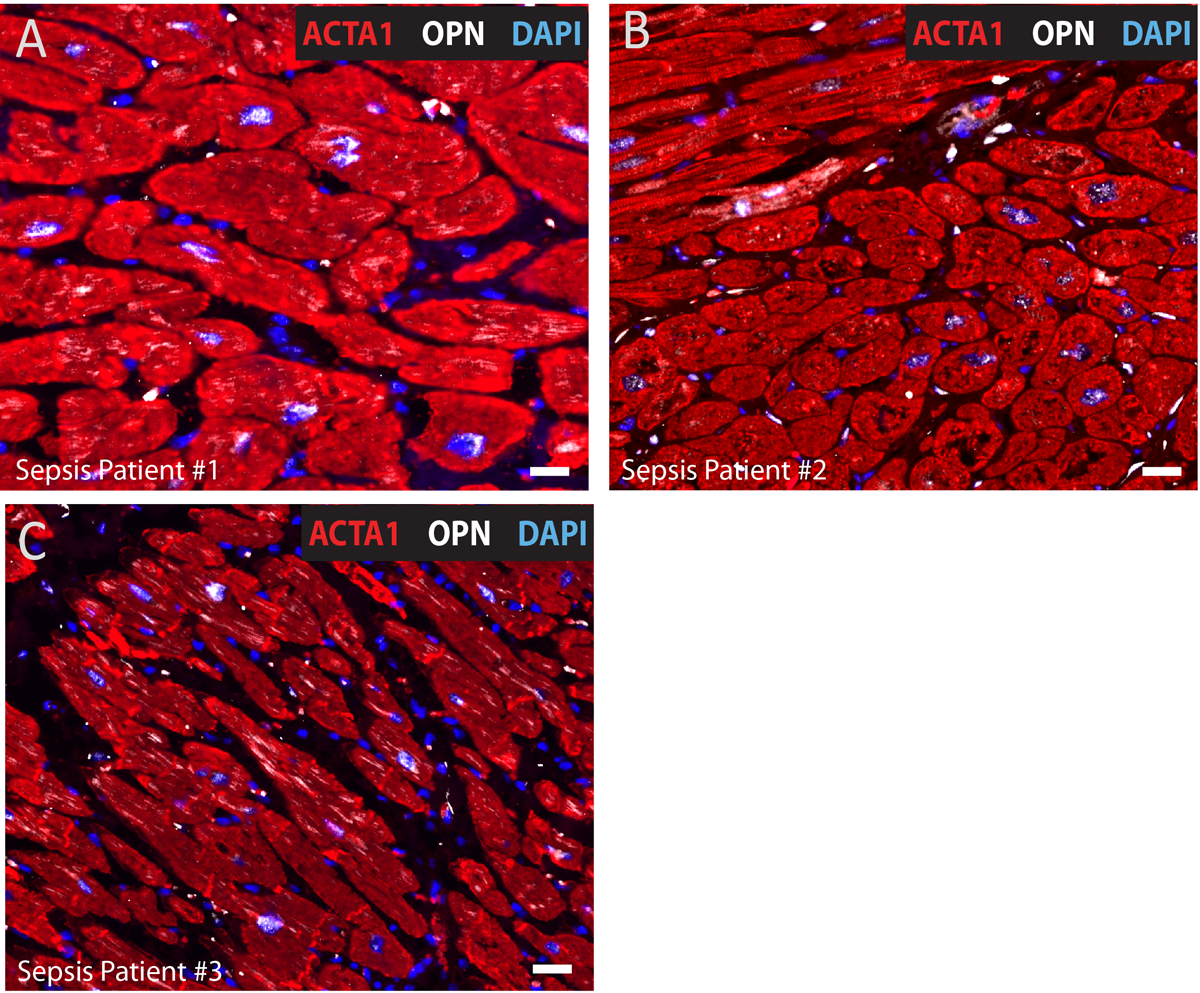

Supplement: FIGURE S2 — Nuclear OPN expression in failing native hearts of sepsis patients. In 3/3 sepsis patients undergoing a heart transplant, nuclear OPN (shown in white) in ACTA1 positive cardiomyocytes (shown in red) is visible in the failing native hearts. Shown are representative fluorescence images from scanner microscope. Scale bar = 20 μm. [file Image_2.TIF]
