# Supplementary material for: Nuclear Osteopontin Is a Marker of Advanced Heart Failure and Cardiac Allograft Vasculopathy: Evidence From Transplant and Retransplant Hearts
Source: Front Physiol. 2020 Aug 13;11:928. doi: 10.3389/fphys.2020.00928 (PMC7438570; doi:10.3389/fphys.2020.00928)
Supplement: TABLE S1 — Clinical condition of three patients with sepsis at time of 1st heart transplant. CAD: coronary artery disease. [file Table_1.docx]

**Supplemental Table 1:** Clinical condition of three patients with sepsis at time of 1^st^ heart transplant.

| **Patient #** | **Clinical Condition** |
| --- | --- |
| 1 | Mild CAD, dilated cardiomyopathy |
| 2 | No CAD, fibrosis, dilated cardiomyopathy |
| 3 | Mild CAD, amyloid, fibrosis |

CAD: coronary artery disease
